# Pinocembrin Protects Human Brain Microvascular Endothelial Cells against Fibrillar Amyloid-*β*_1−40_Injury by Suppressing the MAPK/NF-*κ*B Inflammatory Pathways

**DOI:** 10.1155/2014/470393

**Published:** 2014-07-23

**Authors:** Rui Liu, Jin-ze Li, Jun-ke Song, Jia-lin Sun, Yong-jie Li, Si-bai Zhou, Tian-tai Zhang, Guan-hua Du

**Affiliations:** ^1^Beijing Key Laboratory of Drug Target and Screening Research, Institute of Materia Medica, Chinese Academy of Medical Sciences & Peking Union Medical College, Beijing 100050, China; ^2^Pharmacy Department, the Affiliated Hospital of Medical College, Qingdao University, Qingdao 266003, China

## Abstract

Cerebrovascular accumulation of amyloid-*β* (A*β*) peptides in Alzheimer's disease (AD) may contribute to disease progression through A*β*-induced microvascular endothelial pathogenesis. Pinocembrin has been shown to have therapeutic effects in AD models. These effects correlate with preservation of microvascular function, but the effect on endothelial cells under A*β*-damaged conditions is unclear. The present study focuses on the *in vitro* protective effect of pinocembrin on fibrillar A*β*_1−40_ (fA*β*_1−40_) injured human brain microvascular endothelial cells (hBMECs) and explores potential mechanisms. The results demonstrate that fA*β*_1−40_-induced cytotoxicity in hBMECs can be rescued by pinocembrin treatment. Pinocembrin increases cell viability, reduces the release of LDH, and relieves nuclear condensation. The mechanisms of this reversal from A*β* may be associated with the inhibition of inflammatory response, involving inhibition of MAPK activation, downregulation of phosphor-IKK level, relief of I*κ*B*α* degradation, blockage of NF-*κ*B p65 nuclear translocation, and reduction of the release of proinflammatory cytokines. Pinocembrin does not show obvious effects on regulating the redox imbalance after exposure to fA*β*_1−40_. Together, the suppression of MAPK and the NF-*κ*B signaling pathways play a significant role in the anti-inflammation of pinocembrin in hBMECs subjected to fA*β*_1−40_. This may serve as a therapeutic agent for BMEC protection in Alzheimer's-related deficits.

## 1. Introduction

Brain microvascular endothelial cells (BMECs) contribute to the formation of the blood-brain barrier (BBB) and are indispensable to the creation and maintenance of brain homeostasis. They are also the early targets of various toxic molecules, such as amyloid-*β* peptides (A*β*) and reactive oxygen species (ROS), of neurodegeneration [1, 2]. Recent findings demonstrate that fibrillar A*β* (fA*β*) accumulates not only in the brain parenchyma but also at sites in the cerebrovasculature, particularly around arterioles and capillaries of the cerebral cortex and leptomeninges, resulting in cerebral amyloid angiopathy (CAA) [3, 4]. CAA is closely correlated with Alzheimer's disease (AD) and affects 80–90% of AD patients [4–6]. The pathology of microvascular CAA is associated with cerebrovascular dysfunction, including destruction of the blood-brain barrier (BBB) and enhancement in vessel-associated inflammation [4, 6–8].

In AD, cerebrovascular amyloid deposition primarily comprises aggregated A*β*. Cerebrovascular deficiencies involved in CAA are paralleled by A*β*-mediated responses in cultured endothelial cells [9–11], indicating that A*β* can elicit cerebrovascular deficits that contribute to disease progression. In particular, A*β* treatment of endothelial monolayers augments monocyte adhesion and subsequent transendothelial migration [12–14], reduces endothelial antioxidant efficacy [15–17], stimulates inflammatory responses [18], and increases endothelial permeability [11, 19, 20]. In this way, endothelial damage appears to be associated with the activation of multiple types of signal transduction. A*β* stimulates the production of ROS, which may be important signaling molecules in endothelial pathological events, especially inflammation [21]. ROS may act as second messengers through the activation of many intracellular signaling pathways, such as mitogen-activated protein kinases (MAPKs) and transcription factors, notably nuclear factor-kappa B (NF-*κ*B). The MAPKs are a group of serine and threonine kinases. They regulate gene expression by modulating transcription factors such as NF-*κ*B [22]. They have also been implicated in inflammatory and cell death pathways on cerebral damage [23, 24]. The NF-*κ*B signal pathway, which is activated by ROS-dependent mechanisms [25, 26], also plays an important role in the gene expression of a large number of proinflammatory cytokines. In this way, the hope of counteracting these deleterious events might involve strategies aimed at interrupting the oxidant and inflammatory cascades.

**Figure 1:**
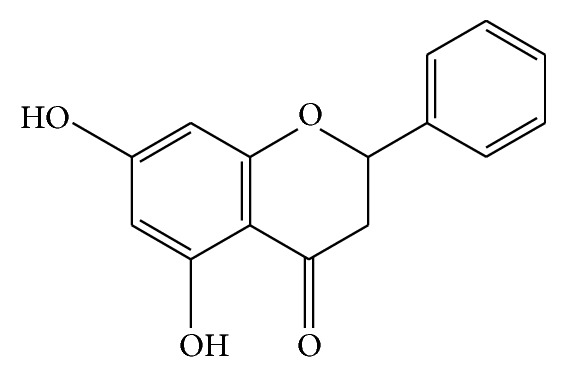
Chemical structure of pinocembrin.

Pinocembrin (5,7-dihydroxyflavanone, Figure 1) is a flavonoid abundant in propolis. It can be extracted as a pure compound and has been shown to be effective in the protection of brain injury from ischemic and A*β* impairment. In 2008, it was approved by the State Food and Drug Administration of China for the treatment of stroke. Pinocembrin was shown to protect against ischemic injury and reduce the area of cerebral infarction in ischemia models via neurovascular protection by decreasing the severity of oxidative damage and inhibiting inflammatory responses [27–29]. It was also demonstrated that pinocembrin protected the BBB from ischemic injury by mitigating ultrastructural damage, reducing permeability, improving microvascular blood flow, and protecting BMECs from oxygen-glucose deprivation/reoxygenation-induced toxicity [30]. Recently, pinocembrin was found to alleviate cognitive deficits in intracerebroventricular A*β*-injected and A*β*-precursor protein (APP)/presenilin 1 (PS1) double transgenic AD mouse models [31, 32]. It has also been shown to attenuate cerebral degeneration in AD by inhibiting inflammatory pathways mediated by the receptor for advanced glycation end products and to be involved in the preservation of the microvascular function, maintenance of the BBB integrity, and reduction of inflammatory mediator levels [31, 32]. Relative studies have demonstrated beneficial effects of pinocembrin in endothelial cells, such as the improvement of the biological functions of endothelial progenitor cells and the suppression of vascular endothelial growth factor-induced angiogenesis in the mouse aortic ring [33, 34]. Although previous studies have suggested that pinocembrin may show protection in endothelial cells from insults, no preexisting study has reported the direct effect of pinocembrin on brain microvascular endothelial cells mediated by A*β*.

Therefore, current* in vitro* studies are yet to be conducted to investigate the effect of pinocembrin on human BMECs (hBMECs) in the fA*β*_1–40_ damaged condition and explore its mechanism during inflammatory processes.

## 2. Material and Methods

### 2.1. Cell Culture and Treatment

hBMECs were purchased from ScienCell Research Laboratories (ScienCell Research Laboratories, Carlsbad, CA, USA). The hBMECs were cultured in endothelial cell complete medium (ScienCell Research Laboratories, Carlsbad, CA, USA) in a 37°C incubator with 5% CO_2_, according to the supplier's recommendations. Experiments were conducted within cell passages 4–6. At these passages, cells displayed a cobblestone appearance, which is morphologically normal for endothelial cells. All treatments were performed after the hBMECs were 60–70% confluent.

Synthetic fA*β*_1–40_ was purchased from Sangon Biotech Company (Shanghai, China) and dissolved in water to make a stock solution of 0.1 mM to foster the fibrillization state, as previously reported [35, 36]. Pinocembrin (purity > 99%) was synthesized by the Institute of Materia Medica of the Chinese Academy of Medical Sciences. It was first dissolved in DMSO at 100 mM and then diluted in endothelial cell medium at 30.0 *μ*M, 10.0 *μ*M, and 3.0 *μ*M. Different concentrations of pinocembrin were added at the start of fibrillar fA*β*_1–40_ injury, and then both were incubated for 24 h. hBMECs were randomly divided into groups: (1) control group, (2) control group treated with 3 *μ*M, (3) control group treated with 10.0 *μ*M, (4) control group treated with 30 *μ*M pinocembrin for 24 h, (5) fA*β*_1–40_ group treated with 20 *μ*M fA*β*_1–40_ for 24 h, (6) fA*β*_1–40_ group treated with 3 *μ*M, (7) 10 *μ*M, and (8) fA*β*_1–40_ group treated with 30.0 *μ*M pinocembrin for 24 h.

### 2.2. MTS Assay for Cell Viability

Cell survival was evaluated using MTS [3-(4,5-dimethylthiazol-2-yl)-5-(3-carboxymethoxyphenyl)-2-(4-sulfophenyl)-2H-tetrazolium, inner salt] assay (Promega, Madison, WI, USA) according to the manufacturer's protocol and detected in a SpectraMax Plus microplate reader (Molecular Devices Corp., Sunnyvale, CA, USA).

### 2.3. Lactate Dehydrogenase (LDH) Release Assay

LDH released from hBMEC-compromised membranes was determined using a CytoTox-ONE Homogeneous Membrane Integrity Assay (Promega, Madison, WI, USA) according to the manufacturer's instructions. Briefly, cells were grown to 70% confluence in 96-well culture plates and were exposed to fA*β*_1–40_, pinocembrin, or both for the indicated lengths of time. Then 50 *μ*L culture supernatants were transferred to a separate assay plate, leaving behind the cells that were used for Hoechst 33343 and ROS detections. Then 50 *μ*L of CytoTox-ONE Reagent was added to each well, and the contents of the plates were mixed for 30 s. The LDH assay was allowed to proceed at room temperature for 10 min prior to an addition of 25 *μ*L/well stop solution containing 10% sodium dodecyl sulfate. The contents of the wells were mixed by shaking the plates for 10 s prior to measurement of resorufin fluorescence (560 nm excitation/590 nm emission).

### 2.4. Hoechst 33342 and DCFH_2_-DA Staining Assay

Nuclear change and intracellular ROS level were measured in hBMECs using Hoechst 33342 (Dojindo Laboratory, Kumamoto, Japan) and DCFH_2_-DA (2′,7′-dihydrodichlorofluorescein diacetate, Sigma Chemical Co., St. Louis, MO, USA) staining, respectively. Nuclei were labeled with 5 *μ*g/mL of Hoechst 33342 at 37°C for 10 min after the fA*β*_1–40_ injury or pinocembrin treatment. ROS was measured based on the oxidation of DCFH_2_-DA to 2′,7′-dichlorofluorescein, and DCFH_2_-DA was added to the culture plates at a final concentration of 5 *μ*M at 37°C for 40 min. The intensity of fluorescence was detected and analyzed by a Cellomics ArrayScan V^TI^ HCS Reader (Cellomics Inc., Pittsburgh, PA, USA) provided with the Morphology Explorer BioApplication software. The images were acquired using the 386/23 nm excitation/460/40 nm emission and 485/20 nm excitation/535/50 nm emission filters, respectively. Nuclear change and ROS level were quantified by the value of average fluorescent intensity [31].

### 2.5. Intracellular Superoxide Dehydrogenase (SOD) and Glutathione Peroxidase (GSH-Px) Assay

After the fA*β*_1–40_ injury and pinocembrin treatment, hBMECs were collected and crushed by sonication (60 W at 0.5 s intervals for 10 min). The cell lysate was centrifuged at 10,000 g for 15 min, and the supernatant was used to measure the activities of SOD and GSH-Px using a WST-1 based SOD inhibition kit (Dojindo Laboratory, Kumamoto, Japan) and a GSH-Px detection kit (Jiancheng Bioengineering, Nanjing, China), respectively. The solutions in each well were added according to the manufacturer's protocols. The absorbance of the endpoint reactions was measured using a SpectraMax Plus microplate reader (Molecular Devices Corp., Sunnyvale, CA, USA). The relative SOD inhibition of each sample was calculated using the following equation: {[(*A*1 − *A*3)−(*As* − *A*2)]/(*A*1 − *A*3)} × 100, where *A*1, *A*2, *A*3, and *As* were the absorbance at 440 nm for the uninhibited test, blank sample, blank reagent, and sample, respectively [37]. GSH-Px activity was determined by quantifying the rate of oxidation of reduced GSH to oxidized GSH by H_2_O_2_ and catalyzed by GSH-Px. One unit of GSH-Px was defined as the amount that could reduce the level of GSH at 412 nm by 1 *μ*M in 1 min per mg of protein.

### 2.6. MAPK Signal Pathways and NF-*κ*B p65 Translocation Assays

The MAPK signal pathways and NF-*κ*B p65 translocation were detected by immunofluorescence assay and quantified on the Cellomics ArrayScan V^TI^ high-content analysis platform. hBMECs were subcultured in black-walled optically clear-bottomed 96-well plates (Corning Life Sciences, Acton, MA, USA). After treatment with fA*β*_1–40_ and pinocembrin as described above, cells were fixed with 4% paraformaldehyde, permeabilized with 0.3% Triton X-100, and then blocked with 3% BSA. The primary antibody mixture containing antiphosphor-ERK1/2 (Thr202/Tyr204) mouse monoclonal (1 : 250, Cell Signaling, Beverly, MA, USA), antiphosphor-p38 (Thr180/Tyr182) rabbit polyclonal (1 : 400, Cell Signaling), anti-phosphor-MAPKAP kinase-2 (MK2) (Thr334) rabbit polyclonal (1 : 200, Cell Signaling), anti-phosphor-SAPK/JNK (Thr183/Tyr185) mouse monoclonal (1 : 400, Cell Signaling), anti-phosphor-c-Jun (Ser73) mouse monoclonal (1 : 200, Cell Signaling), or anti-NF-*κ*B p65 rabbit polyclonal (1 : 250, Invitrogen, Carlsbad, CA, USA) antibodies in PBS was incubated at 4°C overnight. After washing with PBS, the cells were incubated with corresponding AlexaFluor 488 or 546-conjugated goat anti-rabbit/mouse secondary antibodies (1 : 500, Invitrogen, Carlsbad, CA, USA) at room temperature for 1 h. The fluorescence images were acquired using the Cellomics ArrayScan V^TI^ HCS Reader provided with the Cytoplasm to Nucleus Translocation BioApplication [31, 38].

Briefly, images were acquired in independent channels with fixed exposure times. Based on the Hoechst nuclear staining, a nuclear region mask was created and used to quantify nuclear protein distribution. By expanding the nuclear region mask, a concentric ring was generated and used as an approximation of the cytosolic compartment. Cytosolic and nuclear staining intensities were normalized to total nuclear region and cytosolic ring area, allowing for the quantification of protein translocation between the nucleus and cytosol for phosphor-ERK1/2, phosphor-p38, phosphor-MK2, phosphor-SAPK/JNK, and NF-*κ*B p65. The capacity of translocation of the four proteins was illustrated by the value of Mean_CircRingAvgIntenDiff. For the c-Jun detection, nuclear fluorescence intensity was acquired and calculated as the value of protein expression.

### 2.7. Western Blot Analysis

The hBMECs were grown in 10 cm cell culture dishes, exposed to fA*β*_1–40_ plus pinocembrin at the indicated concentrations, and washed twice with PBS before harvesting proteins using ice-cold lysis buffer (50 mM Tris-HCl, pH 7.4, 20 mM EDTA, 0.1% sodium dodecyl sulfate, 100 mM NaCl, 1% NP-40, 0.5% sodium deoxycholate, 50 mM sodium fluoride, 1 mM sodium orthovanadate, 1 mM PMSF, 2 mM sodium pyrophosphate, 1 *μ*g/mL pepstatin A, 100 *μ*g/mL leupeptin, and 1 × protease inhibitor cocktail, Roche Molecular Biochemicals, Indianapolis, IN, USA). The lysates were centrifuged and the resulting supernatant was collected for detection. The samples were separated by SDS-PAGE electrophoresis and transferred to nitrocellulose membranes. The membranes were blocked in Tris-buffered saline and Tween-20 (TBST, pH 7.6) containing 5% nonfat dry milk powder at room temperature for 2 h. The blocks were probed with anti-I*κ*B (inhibitor of kappaB) *α* antibody (1 : 500), anti-phosphor-IKK (I kappaB kinase) *α* antibody (1 : 1000), anti-IKK*α* antibody (1 : 1000), anti-phosphor-IKK*β* antibody (1 : 1000), and anti-IKK*β* antibody (1 : 1000) (Cell signaling, Beverly, MA, USA), respectively, and then with horseradish peroxidase-labeled secondary antibodies (1 : 2000, ZSGB-Bio, Beijing, China). Each membrane was stripped and reprobed with mouse anti-actin antibody which served as a loading control. Relative optical densities and areas of bands were quantified using an imaging densitometer.

### 2.8. ELISA Assay for Tumor Necrosis Factor *α* (TNF-*α*), Interleukin-1*β* (IL-1*β*), and Interleukin-6 (IL-6)

Group divisions and treatments were as described above. The culture medium was collected and centrifuged for 10 min at 4°C to eliminate the cell debris. The proinflammatory cytokines of TNF-*α*, IL-1*β*, and IL-6 in culture medium were measured by ELISA assays. Quantitative levels were measured according to the manufacturer's instructions (Jiameinuosi Biotech, Beijing, China). The optical density was measured at 450 nm, and values were calculated with reference to standard curves.

### 2.9. Statistical Analysis

All data are represented as the mean ± the standard error of the mean (SEM). Comparisons were performed using one-way analysis of variance (ANOVA), and multiple comparisons were performed using post-hoc least significant difference comparisons. A *P* value of <0.05 was considered statistically significant.

## 3. Results

### 3.1. Pinocembrin Protects hBMECs from fA*β*_1–40_-Induced Cytotoxicity

**Figure 2:**
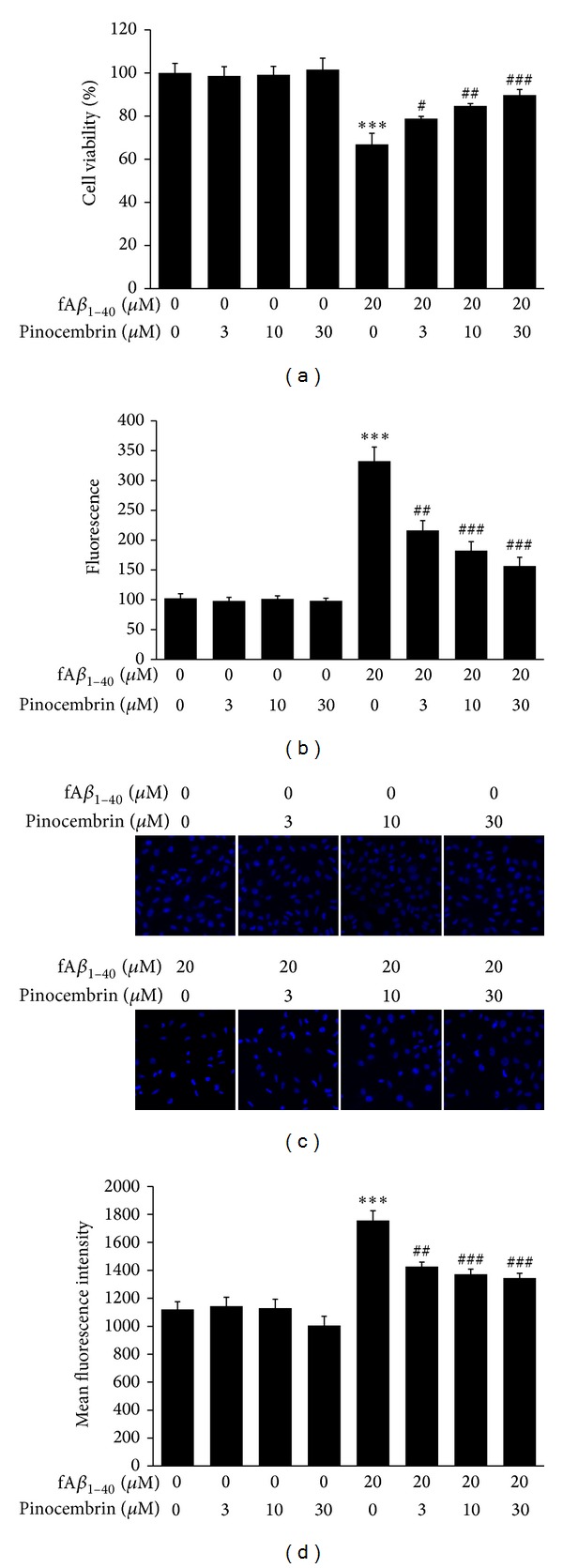
Cytoprotective effects of pinocembrin on hBMECs against fA*β*_1–40_-induced toxicity. (a) Pinocembrin increases cell viability as evaluated by MTS assay. (b) Pinocembrin decreases the levels of extracellular LDH released from hBMECs against fA*β*_1–40_-induced toxicity. (c) Representative images of nuclei stained by Hoechst 33342. Pinocembrin attenuates nuclear damage in hBMECs in the presence of fA*β*_1–40_ (×20). (d) Pinocembrin inhibits nuclear mean fluorescence intensity in hBMECs against fA*β*_1–40_-induced toxicity. Data are expressed as means ± SEM, *n* = 6, ***P < 0.001 versus control, ^#^ *P* < 0.05, ^##^ *P* < 0.01, ^###^ *P* < 0.001 versus fA*β*_1–40_.

In the present study, the direct protective effects of pinocembrin on hBMECs against fA*β*_1–40_-induced toxicity were examined in three cytotoxicity assays. In the MTS assay, cell viability was found to be significantly decreased in the presence of 20 *μ*M fA*β*_1–40_ in hBMECs (Figure 2(a),  *P* < 0.001). Pinocembrin increased cell viability at 3.0 *μ*M, 10.0 *μ*M, and 30.0 *μ*M after exposure to 20 *μ*M fA*β*_1–40_ in a dose-dependent manner (*P* < 0.05,  *P* < 0.01,   *P* < 0.001). Pinocembrin did not show significant effects on hBMECs without fA*β*_1–40_ treatment at any of the concentrations evaluated here.

A similar effect was seen in the LDH release assay. Concentrations of 20 *μ*M fA*β*_1–40_ produced significant enzyme leakage from hBMECs (Figure 2(b),  *P* < 0.001), and the intensity of fluorescence based on the release of LDH in cells treated with pinocembrin decreased significantly at 3.0 *μ*M, 10.0 *μ*M, and 30.0 *μ*M in a dose-dependent manner (*P* < 0.01,  *P* < 0.001). Pinocembrin did not affect the release of LDH from hBMECs without fA*β*_1–40_ treatment at the same concentrations.

The cytoprotective effects of pinocembrin were confirmed in nuclear changes by Hoechst 33342 staining as well. Control hBMECs were uniformly stained with faint blue fluorescence. In contrast, hBMECs treated with fA*β*_1–40_ showed denser nuclei with more intense fluorescence indicating nuclear shrinkage or condensation which is one of the early signs of damage (*P* < 0.001, Figures 2(c) and 2(d)). These cytotoxic effects were alleviated in both nuclear morphological condensation and fluorescence through the treatment with pinocembrin at 3.0 *μ*M, 10.0 *μ*M, and 30.0 *μ*M (*P* < 0.01,  *P* < 0.001). Pinocembrin detected at the same concentrations did not damage the nuclei of control cells.

Based on the above results, pinocembrin at the determined concentrations of 3.0 *μ*M, 10.0 *μ*M, and 30.0 *μ*M is found to significantly increase the viability of cells, decrease the level of LDH, and relieve the injury of nucleus injured by fA*β*_1–40_. Pinocembrin detected at the same concentrations did not show effects in these cytotoxicity assays. Due to the dose-dependent manner in which pinocembrin is found to act, concentrations ranging from 3.0 *μ*M to 30.0 *μ*M are selected for further investigation in hBMECs in the presence to fA*β*_1–40_.

### 3.2. Pinocembrin Cannot Remarkably Regulate the Redox Imbalance of hBMECs against fA*β*_1–40_-Induced Toxicity

**Figure 3:**
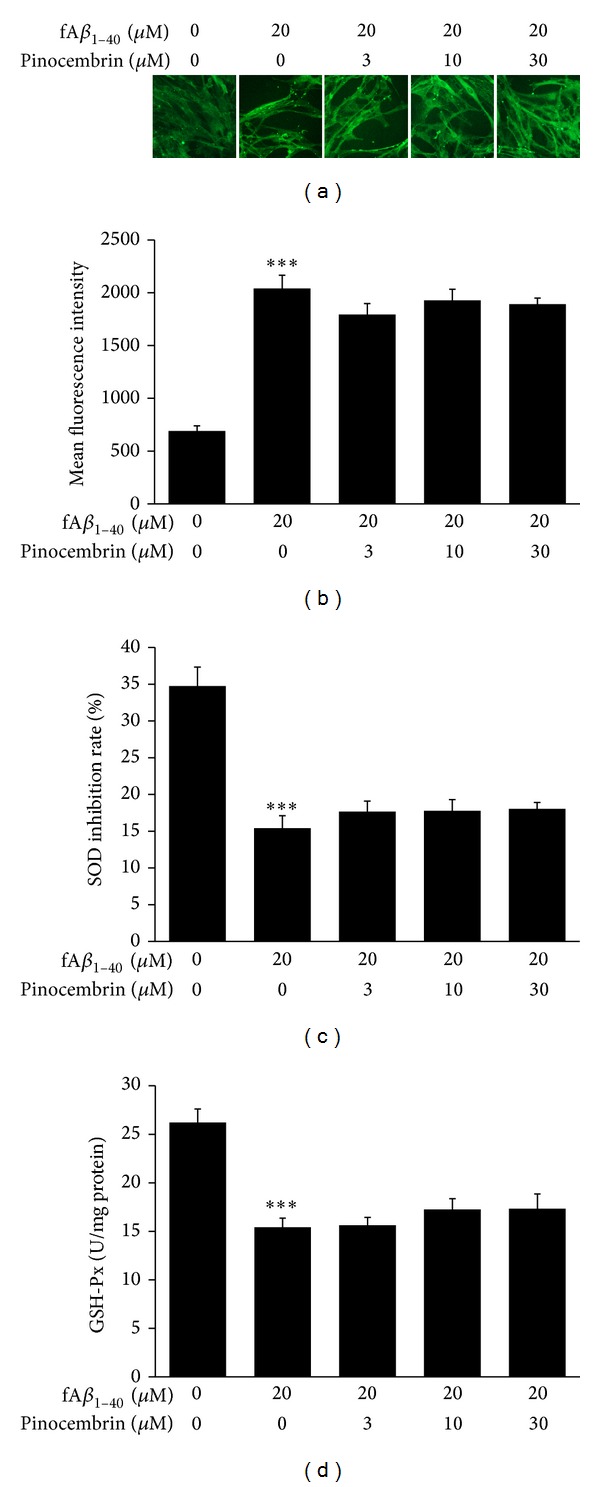
Effects of pinocembrin on redox imbalance of hBMECs against fA*β*_1–40_-induced toxicity. (a) Representative images of intracellular ROS stained by DCFH_2_-DA. Pinocembrin cannot decrease the generation of ROS in hBMECs in the presence of fA*β*_1–40_ (×20). (b) Intracellular mean fluorescence intensity was assessed based on the DCF fluorescence on the ArrayScan HCS Reader with the Morphology Explorer BioApplication. Pinocembrin does not decrease mean fluorescence intensity in hBMECs against fA*β*_1–40_-induced toxicity. (c) Pinocembrin does not improve the SOD activity in hBMECs against fA*β*_1–40_-induced toxicity. (d) Pinocembrin does not increase the GSH-Px activity in hBMECs against fA*β*_1–40_-induced toxicity. Data are expressed as means ± SEM, *n* = 6, ***P < 0.001 versus control.

A*β* exerts toxicity against the endothelial cells of the brain via enhanced ROS production and redox imbalance [37, 39]. In this study, fA*β*_1–40_ increased endogenous ROS generation in hBMECs by about 2.95-fold (*P* < 0.001, Figures 3(a) and 3(b)). fA*β*_1–40_ also reduced endothelial antioxidant efficacy through decreasing GSH-Px and SOD activities, two markers of oxidative stress, to 43.36% and 58.82%, respectively (*P* < 0.001, Figures 3(c) and 3(d)). These effects indicated a severe redox imbalance in this endothelial cell model. However, pinocembrin neither decreased the ROS generation nor increased GSH-Px or SOD activity in the present model at the concentrations evaluated here, suggesting that pinocembrin cannot exert sufficient effects on amelioration of the antioxidative ability of hBMECs subjected to fA*β*_1–40_-induced toxicity.

### 3.3. Pinocembrin Inhibits the MAPK Pathways in hBMECs against fA*β*_1–40_-Induced Toxicity

**Figure 4:**
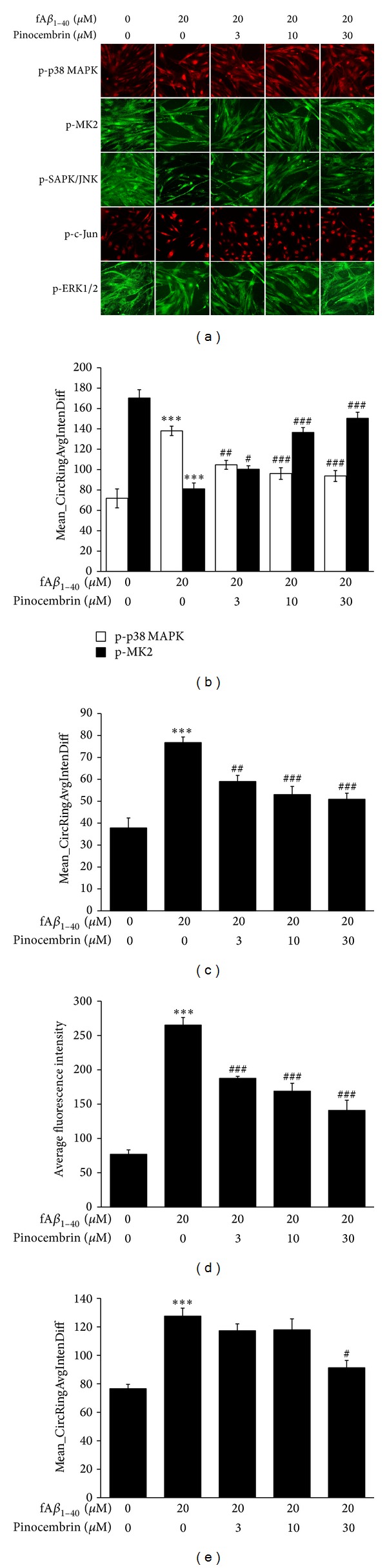
Effects of pinocembrin on MAPK pathways of hBMECs against fA*β*_1–40_-induced toxicity. (a) Images of phosphor-p38, phosphor-MK2, phosphor-SAPK/JNK, phosphor-c-Jun, and phosphor-ERK1/2 were acquired on the ArrayScan HCS Reader using the Cytoplasm to Nucleus Translocation BioApplication (×20). (b), (c), and (e) Values of Mean_CircRingAvgIntenDiff describe the capacity translocation of cytosolic phospho-p38, phospho-SAPK/JNK, and phospho-ERK1/2 to the nucleus and nuclear phospho-MK2 to the cytoplasm. (d) Nuclear average fluorescence intensity illustrates the expression of phosphor-c-Jun. Data are expressed as means ± SEM, *n* = 6, ***P < 0.001 versus control, ^#^ *P* < 0.05, ^##^ *P* < 0.01, ^###^ *P* < 0.001 versus fA*β*_1–40_.

MAPKs are regulated by ROS in endothelial cells to express the proinflammatory phenotype through the phosphorylation activation and the subsequent nuclear transduction [40–42]. Here, in control hBMECs, basal levels of phosphor-p38 and phosphor-MK2 were significantly confined to the cytosolic and nuclear compartment, showing a low and a high Mean_CircRingAvgIntenDiff value, respectively. Depending on stimulus of fA*β*_1–40_, phosphor-p38 and phosphor-MK2 translocation were activated, as illustrated by the significant increase and a marked decrease in Mean_CircRingAvgIntenDiff values, respectively (*P* < 0.001, Figures 4(a) and 4(b)). Pinocembrin treatment significantly inhibited the p38 MAPK pathway. The translocation of cytosolic phosphor-p38 to the nucleus and nuclear phosphor-MK2 to the cytoplasm was significantly inhibited at concentrations of 3.0 *μ*M, 10.0 *μ*M, and 30.0 *μ*M in a dose-dependent manner (*P* < 0.05, *P* < 0.01, *P* < 0.001).

The basal level of phosphor-SAPK/JNK was confined to the cytosolic compartment, shown as a low Mean_CircRingAvgIntenDiff value in control hBMECs. Similarly, its downstream phosphor-c-Jun was seen in low average fluorescence intensity in the nucleus. After fA*β*_1–40_ treatment, phosphor-SAPK/JNK translocation was promoted by a significant increase in Mean_CircRingAvgIntenDiff values (*P* < 0.001, Figures 4(a) and 4(c)). The level of phosphor-c-Jun increased by 3.6-fold, as indicated by average intensity of fluorescence in the nucleus (*P* < 0.001, Figures 4(a) and 4(d)). Pinocembrin treatment was found to significantly inhibit the SAPK/JNK pathway. The translocation of cytosolic phosphor-SAPK/JNK to the nucleus and the upregulation of phosphor-c-Jun were inhibited at concentrations of 3.0 *μ*M, 10.0 *μ*M, and 30.0 *μ*M in a dose-dependent manner (*P* < 0.01, *P* < 0.001, Figures 4(a), 4(c), and 4(d)). Similarly, phosphor-ERK1/2 translocation was activated by showing a significant increase in the Mean_CircRingAvgIntenDiff value in fA*β*_1–40_-treated hBMECs (*P* < 0.001, Figures 4(a) and 4(e)), and pinocembrin was shown to significantly inhibit translocation of cytosolic phosphor-ERK1/2 to the nucleus at 30.0 *μ*M (*P* < 0.05).

### 3.4. Pinocembrin Regulates NF-*κ*B Signal Pathway and Inhibits the Release of Proinflammatory Cytokines in hBMECs against fA*β*_1–40_-Induced Toxicity

**Figure 5:**
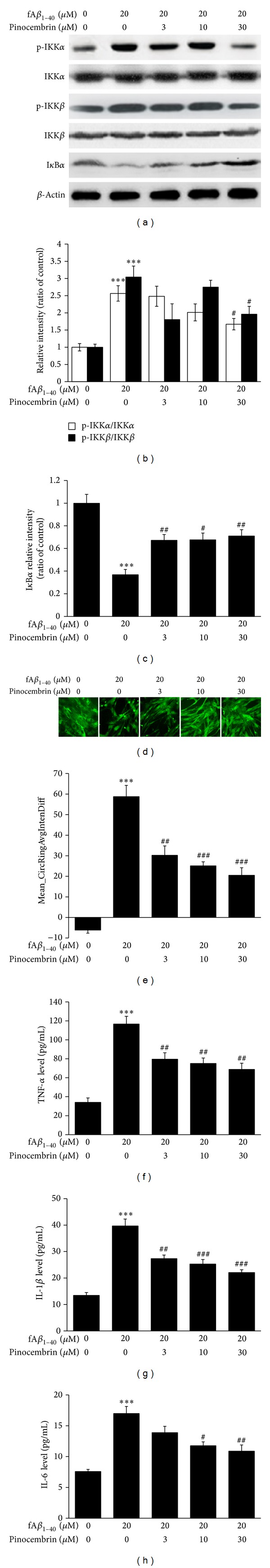
Effects of pinocembrin on the NF-*κ*B signal activation and the release of proinflammatory cytokines of hBMECs against fA*β*_1–40_-induced toxicity. (a) Representative immunoblots for phosphor-IKK*α*, IKK*α*, phosphor-IKK*β*, IKK*β*, and I*κ*B*α* in hBMECs extracts. (b) Quantitative analysis of these proteins. (d) Images of NF-*κ*B p65 translocation from cytoplasm to nucleus analyzed on the ArrayScan HCS Reader (×20). (e) The value of Mean_CircRingAvgIntenDiff describing the translocation capacity of cytosolic phospho-p65 to the nucleus. (f), (g), and (h) Levels of TNF-*α*, IL-1*β* and IL-6 in hBMECs culture supernatant after exposure to fA*β*_1–40_. Data are expressed as means ± SEM, *n* = 6, ***P < 0.001 versus control, ^#^ *P* < 0.05, ^##^ *P* < 0.01 versus fA*β*_1–40_.

Activation of NF-*κ*B is a central event in the inflammatory response, and optimal activation of NF-*κ*B requires the release of p65 from I*κ*B following the phosphorylation of the I*κ*B proteins by a complex of I*κ*B kinases. fA*β*_1–40_ was found to induce upregulation in phosphorylation levels of IKK*α* and IKK*β* (*P* < 0.001, Figures 5(a) and 5(b)), decrease the levels of I*κ*B*α* (*P* < 0.001, Figures 5(a) and 5(c)), and increase the NF-*κ*B p65 distribution in nucleus (*P* < 0.001, Figures 5(d) and 5(e)). The levels of TNF-*α*, IL-1*β*, and IL-6 in culture medium were increased as well (*P* < 0.001, Figures 5(f)–5(h)). Compared with the fA*β*_1–40_-treated hBMECs, quantitative analysis revealed statistically significant downregulation of phosphorylation levels of IKK*α* and IKK*β* in 30 *μ*M pinocembrin-treated group (*P* < 0.05). Besides for this effect, pinocembrin markedly suppressed the activation of NF-*κ*B signal transduction through blockage of the degradation of I*κ*B*α* and inhibition of the nuclear translocation of NF-*κ*B p65 at concentrations of 3.0 *μ*M, 10.0 *μ*M, and 30.0 *μ*M in a dose-dependent manner (*P* < 0.05, *P* < 0.01, *P* < 0.001, Figures 5(a) and 5(c)–5(e)). The levels of TNF-*α*, IL-1*β*, and IL-6 in culture medium were also significantly decreased by pinocembrin in the fA*β*_1–40_-treated hBMEC model (*P* < 0.05, *P* < 0.01, *P* < 0.001, Figures 5(f)–5(h)).

## 4. Discussion

As an extension of previous research, the present study clarified the beneficial effects of pinocembrin on AD-associated microvascular endothelial pathology. The present findings indicate that pinocembrin can protect hBMECs from fA*β*_1–40_-induced toxicity. In these effects, pinocembrin increases cell viability, reduces the amount of LDH release, relieves nuclear condensation, inhibits the MAPK pathways, relieves I*κ*B*α* degradation, blocks NF-*κ*B p65 nuclear translocation, and reduces the levels of extracellular proinflammatory cytokines. In addition, pinocembrin can inhibit phosphor-IKK activation modestly. However, pinocembrin does not show remarkable effects on the regulation of redox imbalances. Pinocembrin's ability to protect microvascular endothelial cells from fA*β*_1–40_ mainly contributes to anti-inflammation.

The microvascular endothelial cells of the brain form a highly specialized endothelial tissue that serves as the BBB. These cells appear to be a primary target and an important responsive component of cerebral inflammation in AD [1, 2]. Cerebrovascular A*β* deposition plays a role in the progression of AD. Fibrillar A*β* accumulates at sites in the cerebrovasculature, particularly around arterioles and capillaries of the cerebral cortex and leptomeninges [3, 4]. This phenomenon is present in more than 80% of AD patients, and cerebrovascular amyloidosis is causally involved in the development of neurodegeneration in this disease [42].

Given experimental data reporting the contradictory findings of A*β* toxicity in endothelial cell culture [9, 10, 43], the present work first involved confirmation of whether fA*β*_1–40_ treatment causes any significant cell death in the present experimental paradigm. In line with the decreased viability of hBMECs in the presence of fA*β*_1–40_, the increased release of LDH and the injury of nuclei were observed in the same manner. These results demonstrate that fibrillar fA*β*_1–40_ is directly toxic to hBMECs.

Herein, the effective administration conditions for pinocembrin were screened using both control and fA*β*_1–40_-injured hBMECs in the first step. Pinocembrin at the determined optimal concentrations of 3.0 *μ*M, 10.0 *μ*M, and 30.0 *μ*M is found to significantly increase the viability of cells injured by fA*β*_1–40_. Results of the decrease of LDH release and the relief of nuclear injury also indicate the protective effects of pinocembrin on this process. It is here confirmed that there are no differences among the pinocembrin treatments in control cells, indicating that pinocembrin has no toxic effect under basal conditions.

Oxidative stress is implicated in AD pathology. Excessive generation of ROS within endothelial cells in response to A*β* leads to oxidative stress and cellular injury [39].* In vitro* studies have revealed that oxidative stress results in dysfunction of the endothelial cell, destroying the integrity of the vascular barrier and leading to increased endothelial permeability, mitochondrial dysfunction, generation of cytokines, chronic inflammatory processes, and amyloid deposition in blood vessels, which are involved with the imbalance of endothelial transductions during the pathogenesis of Alzheimer's deficits [11, 19, 44–48]. A strategy involving neuroprotective properties is here recommended for reduction of the severity of oxidative injury and maintaining the integrity of the BBB for the treatment of brain damage.

Pinocembrin is a flavonoid so that it might be thought to be effective in quenching free radicals. Here, pinocembrin merely produced a slight increase in the effectiveness of the antioxidant defense system of hBMECs when subjected to fA*β*_1–40_. Pinocembrin was reported to possess a limited antioxidative effect in ischemia models [28, 29], but it is not found to produce sufficient effects on the regulation of the redox imbalance under the conditions that are rich in A*β* [31, 32]. In general, the cytoprotective capacity of flavonoids against different insults has been mainly attributed to their antioxidant potency [49, 50]. Nonetheless, cytoprotection of flavonoids was no more defined to be correlated with the antioxidation potency [51]. The report that the hydroxy substitutions in the* A*-ring (*C*5 and* C*7) and in position* C*3 (*C*-ring) of the flavones would be necessary to afford neuroprotection indicated that the structural requirements for cytoprotection are different from those that afford antioxidant capacity [52]. Additionally, many flavonoids show an important pharmacological effect on modulating the activities of protein kinases, lipid kinases, and enzymes of mitochondrial respiratory chain independent of their antioxidant capacity [53–55]. Therefore, although antioxidation is not involved in the major mechanisms that prevent A*β*-mediated toxicity, pinocembrin may act synergistically with other crucial mechanisms for the treatment in our experimental model.

The MAP kinase family is correlated with activation of intracellular signal events during the pathological process of AD. To date, at least three major MAPK cascades have been described that involve the activation of ERK, SAPK/JNK, and p38 MAPK in the brain. The ERK cascade is mostly responsive to mitogenic and differentiation stimuli, whereas the JNK and p38 MAPK pathways are preferentially activated by proinflammatory cytokines and extracellular stress [56] and contribute to the regulation of synaptic function, the BBB permeability, inflammatory response, and apoptotic process [57–59]. One type of stress that induces potential activation of MAPK pathways is the oxidative stress caused by ROS. In case MAPK-signaling pathways are activated, several inflammatory cytokines are further overproduced and released [56].

MAPK activation may be secondary to many different extracellular stimuli, but the series of phosphorylation cascade activation events are critical to the responses. Our results showed that fA*β*_1–40_ exposure induced activation of ERK1/2, p38 MAPK/MK2, and SAPK/JNK-c-Jun cascades secondary to overproduction of ROS in the hBMECs. This is consistent with the results of the proinflammatory nature of the MAPK pathway [60]. Pinocembrin is found to markedly inhibit the activation of phosphorylated p38 MAPK/MK2 and SAPK/JNK-c-Jun pathways at each tested concentration, and only the high concentration (30 *μ*M) was effective in the blockade of phosphor-ERK1/2 activation. These results are essentially consistent with the finding that pinocembrin modulates transduction of these MAPKs in neuronal and endothelial cells as in previous reports as well [31, 32]. Besides, many molecular components are involved in apoptosis tightly linked to the presence and activation of MAPK family, one of which is the JNK-mediated cytochrome *c* release contributing to caspase-3 activation and the onset of apoptosis [61, 62]. Therefore, these inseparable processes can be inhibited not only by antioxidant treatment but also by MAPK activation inhibition [63–65]. Although pinocembrin does not show strong antioxidative effects through the clearance of ROS, coincided with the reversal from the endothelial injury, it is plausible that pinocembrin treatment attenuates fA*β*_1–40_-induced cytotoxicity, at least in part, through the inhibition of ERK1/2, SAPK/JNK, and p38 cascades. However, in this study, we only determined the effect of pinocembrin on translocation cascades of MAPKs following the phosphorylation. It is worth investigating more precisely in the future the effect of pinocembrin on the phosphorylation levels of MAPK family.

It is evidenced that ROS and MAPK signal pathways are involved in the regulation of NF-*κ*B activation in response to stress. As a redox-sensitive transcription factor, NF-*κ*B is activated via the activation of I*κ*B-kinase complex which then phosphorylates I*κ*B on Ser 32 and Ser 36, resulting in its ubiquitination and subsequent proteasomal degradation as well as the release of NF-*κ*B, which can translocate into the nucleus to activate the transcription of proinflammatory target genes, such as TNF-*α*, IL-1*β*, and IL-6 [66, 67]. The protective effects of pinocembrin against A*β*-stimulated endothelial responses were also mediated by blocking NF-*κ*B signaling pathways. However, different concentrations of pinocembrin were required to inhibit specific inflammatory transduction in these signal pathways. Only 30 *μ*M of pinocembrin was found to significantly decrease the levels of phosphorylation of IKK*α* and IKK*β*, which indicates a slight reduction in the concentration of the I*κ*B-kinase complex degradation of the NF-*κ*B signaling regulated by this compound. Activation of NF-*κ*B needs I*κ*B*α* to be phosphorylated, which then leads to targeted degradation of I*κ*B*α*. The following dissociation of I*κ*B*α* causes the translocation of NF-*κ*B from the cytoplasm to the nucleus where it binds and triggers the inflammatory gene expression. All concentrations of pinocembrin tested in this study significantly attenuated the degradation of I*κ*B*α* and inhibited the nuclear translocation of p65. Although multiple signal molecules are involved in the NF-*κ*B pathway, we suggested that pinocembrin could remarkably inhibit the activation of NF-*κ*B signal transduction by attenuating the degradation of the inhibitory protein I*κ*B*α* and blocking the translocation of NF-*κ*B p65.

The level of proinflammatory cytokines also conduces to evaluate endothelial cell injuries. In response to A*β* stimulus, a series of intracellular signaling cascades are initiated which ultimately lead to activation of inflammation and the release of proinflammatory cytokines in endothelial cells [68]. Many studies demonstrated that some of the proinflammatory cytokines, such as TNF-*α*, IL-1*β*, and IL-6, play a key role in the development and maintenance of inflammation, and this cytokine elevation is associated with neurodegenerative diseases [69]. TNF-*α*, IL-1*β*, and IL-6 may also serve as biomarkers of the NF-*κ*B inflammatory pathway [70]. Considering that the transcription levels of these cytokines are under the control of NF-*κ*B, we investigated whether the reduced secretion levels of TNF-*α*, IL-1*β*, and IL-6 in pinocembrin-treated cells were due to inhibition of NF-*κ*B signaling. In line with the above findings regarding NF-*κ*B transduction, the levels of TNF-*α*, IL-1*β*, and IL-6 in culture medium were significantly decreased by pinocembrin at all tested concentrations. Thus, we suggest that pinocembrin has anti-inflammatory effects against fA*β*_1–40_-induced toxicity, and its mechanisms can be routed through the NF-*κ*B signaling pathways to inhibit the secretion of TNF-*α*, IL-1*β*, and IL-6.

Further, MAPK transductions which have been implicated to be key modulators in inflammatory signaling cascades are associated with activation with NF-*κ*B in the inflammatory response [71–73]. These MAPKs regulate NF-*κ*B activation through I*κ*B*α*/*β* kinase activation which induces I*κ*B degradation [72, 73]. Thus, we deduce that the NF-*κ*B along with MAPKs may participate in the amplifying loop of the inflammatory responses after hBMECs subjected to fA*β*_1–40_, and that the modulations of the three-tiered cascades provide a rationale to evidence the therapeutic effects of pinocembrin against fA*β*_1–40_-mediated toxicity in hBMECs. Previous studies evidenced that the MAPKs especially p38 and JNK have been implicated in the regulation of inflammatory mediators, including the proinflammatory cytokines, which make them potential targets for anti-inflammatory therapeutics [74, 75]. Since p38 and JNK activation in the present study was more responsive to the inhibitory effects of each concentration (from 3 *μ*M to 30 *μ*M) of pinocembrin than ERK that required the high concentration (30 *μ*M), it is assumed that the anti-inflammatory effect of pinocembrin against fA*β*_1–40_ in hBMECs may depend primarily on the inhibition of the p38 MAPK and JNK activation. As another point of view, the relevant results may provide the explanation that due to the secondary effect in the inflammatory loop pinocembrin showed a slight reduction in the phosphorylation levels of IKK*α* and IKK*β*.

**Figure 6:**
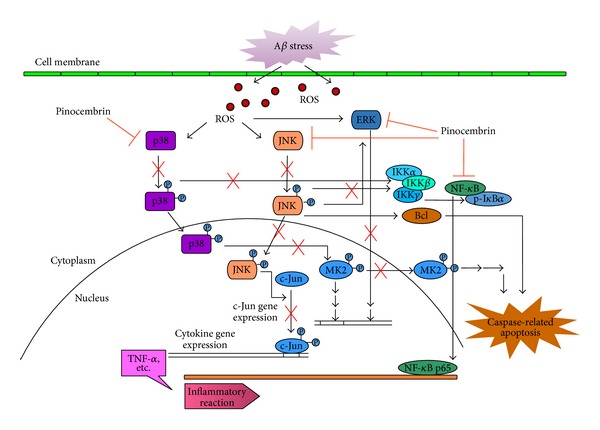
Schematic diagram of the potential mechanisms for the action of pinocembrin against A*β*-induced toxicity in brain microvascular endothelial cells. Pinocembrin suppresses the activation of several subfamilies of MAPK-signaling cascades induced by A*β* injury in hBMECs. Pinocembrin also exerts an anti-inflammatory effect through attenuating the degradation of I*κ*B*α* and blocking the nuclear translocation of NF-*κ*B p65. Pinocembrin shows a slight reduction in the phosphorylation levels of IKK compensatively in the modulation of NF-*κ*B signaling as well.

Taking the results of cytotoxicity assays and inflammatory measurements together, pinocembrin is capable of reducing the fA*β*_1–40_ damage, increasing cell survival, and decreasing the proinflammatory cytokine levels in a consistent and effective manner, suggesting that pinocembrin might affect the regulation of the balance of cell survival and inflammatory response in cerebral endothelial cells. Previous studies have shown the therapeutic role and mechanism of pinocembrin involved in Alzheimer's-related deficits. It has been demonstrated that it is effective in conferring neurovascular protection through maintenance of neuropil ultrastructure and the reduction of glial activation and levels of inflammatory mediators in the brain [31, 32]. Pinocembrin has been shown to promote neurovascular inflammatory pathways against various types of A*β* toxicity [31, 32]. In the present study, the direct recovery of hBMECs by pinocembrin was confirmed and the underlying mechanisms were identified. Three possible intracellular signaling pathways were found to be involved in the modulation of inflammatory responses of pinocembrin in endothelial cells (Figure 6). Firstly, because pinocembrin suppresses activation of several subfamilies of MAPK-signaling cascades induced by A*β* injury in hBMECs in accordance with the inhibition of neurovascular inflammatory pathways, it is here suggested that MAPKs pathway may be one of the mechanisms by which pinocembrin inhibits overproduction of proinflammatory cytokines. Secondly, because modulation of NF-*κ*B activation provides a mean of reducing the generation of inflammatory factors against multiple A*β* insults conducted both in this study and in previous research, it is here suggested that pinocembrin also exerts an anti-inflammatory effect through attenuating the degradation of I*κ*B*α* and blocking the nuclear translocation of NF-*κ*B p65. Thirdly, as the NF-*κ*B along with MAPKs may participate in the amplifying loop of the inflammatory responses in our experiments, pinocembrin shows a slight reduction in the phosphorylation levels of IKK as a compensative modulation in the NF-*κ*B signaling.

## 5. Conclusion

In summary, the present study demonstrates that fA*β*_1–40_-induced cytotoxicity in hBMECs can be rescued by pinocembrin treatment. The endothelial protective effects against fA*β*_1–40_ exhibited by pinocembrin are achieved via anti-inflammation. The mechanisms of this reversal from A*β* may be involved in the inhibition of MAPK activation, the decrease in phosphor-IKK activation, the relief of I*κ*B*α* degradation, the blockage of NF-*κ*B p65 nuclear translocation, and the reduction of proinflammatory cytokine release. Pinocembrin does not show sufficient activity on regulating the redox imbalance. Taken together, the suppression of MAPK and NF-*к*B signaling pathways might play a significant role in the endothelial protection of pinocembrin. In this way, it may serve as a potential therapeutic agent for BMECs in the prevention of Alzheimer's-related deficits.
